# Challenges and lessons learned from two countries using linked administrative data to evaluate the Family Nurse Partnership.

**DOI:** 10.23889/ijpds.v7i3.1833

**Published:** 2022-08-25

**Authors:** Francesca Cavallaro, Rebecca Cannings-John, Fiona Lugg-Widger, Jan van der Meulen, Ruth Gilbert, Eilis Kennedy, Michael Robling, Hywel Jones

**Affiliations:** 1 The Health Foundation; 2 Cardiff University; 3 London School of Hygiene & Tropical Medicine; 4 UCL GOS Institute of Child Health; 5 Tavistock and Portman NHS Foundation Trust

## Objectives

We describe the challenges and lessons learned from two studies using linked administrative data from health, education and social care sectors to evaluate the Family Nurse Partnership (FNP), an intervention supporting adolescent mothers in England(E) and Scotland(S). We present recommendations for studies using linked administrative data to evaluate complex interventions.

## Approach

We constructed two cohorts of all mothers aged 13-19 giving birth in NHS hospitals in England and Scotland between 2010-2016/17 using linkage of mothers and babies in hospital admissions data (E:Hospital Episode Statistics/S:Maternity Inpatient and Day Case), and identified FNP participation through linkage to FNP programme data. We additionally linked to health, educational and social care data for mothers and their babies (E:National Pupil Database/S:eDRIS). We used these data to identify key risk factors for enrolment in the FNP, assess the effect of the FNP on maternal and child outcomes, and determine programme characteristics modifying the effect of the FNP.

## Results

Key challenges: characterising the intervention and usual care, understanding quality of multi-sector data linkage, data access delays, constructing appropriate comparator groups and interpreting outcomes captured in administrative data. Lessons learned: evaluations require detailed data on intervention activity (dates/geography), and assessment of usual care, which are rarely readily available and are time-consuming to gather; data linkage quality is variable/not available, making defining denominators challenging; data access delays impeded on data analysis time; unmeasured confounders not captured in administrative data may prevent generation of an appropriate comparator group. Recommendations: Characteristics informing targeting should be explicitly documented, and could be enhanced using linked primary care data and information on household members (e.g. fathers). Process evaluation and qualitative research could help to provide better understanding of mechanisms of effect.

## Conclusion

Linkage of administrative data presents exciting opportunities for efficient evaluation of large-scale, complex public health interventions. However, sufficient information is needed on programme meta-data, targeting and important confounders in order to generate meaningful results. Study findings should help stimulate exploration with practitioners about how programmes can be improved.

